# A Potential Theragnostic Regulatory Axis for Arthrofibrosis Involving Adiponectin (ADIPOQ) Receptor 1 and 2 (ADIPOR1 and ADIPOR2), TGFβ1, and Smooth Muscle α-Actin (ACTA2)

**DOI:** 10.3390/jcm9113690

**Published:** 2020-11-17

**Authors:** Banu Bayram, Aaron R. Owen, Amel Dudakovic, Louis Dagneaux, Travis W. Turner, Jacob W. Bettencourt, Afton K. Limberg, Meagan E. Tibbo, Mark E. Morrey, Joaquin Sanchez-Sotelo, Daniel J. Berry, Jean-Pierre A. Kocher, Andre J. van Wijnen, Matthew P. Abdel

**Affiliations:** 1 Department of Orthopedic Surgery, Mayo Clinic, Rochester, MN 55905, USA; bayram.banu@mayo.edu (B.B.); owen.aaron@mayo.edu (A.R.O.); dudakovic.amel@mayo.edu (A.D.); dagneaux.louis@mayo.edu (L.D.); turner.travis@mayo.edu (T.W.T.); bettencourt.jacob@mayo.edu (J.W.B.); limberg.afton@mayo.edu (A.K.L.); tibbo.meagan@mayo.edu (M.E.T.); morrey.mark@mayo.edu (M.E.M.); sanchezsotelo.joaquin@mayo.edu (J.S.-S.); berry.daniel@mayo.edu (D.J.B.); 2 Department of Biochemistry & Molecular Biology, Mayo Clinic, Rochester, MN 55905, USA; 3 Department of Health Science Research, Mayo Clinic, Rochester, MN 55905, USA; kocher.jeanpierre@mayo.edu

**Keywords:** arthrofibrosis, stiffness, total knee arthroplasty (TKA)

## Abstract

(1) Background: Arthrofibrosis is a common cause of patient debility and dissatisfaction after total knee arthroplasty (TKA). The diversity of molecular pathways involved in arthrofibrosis disease progression suggest that effective treatments for arthrofibrosis may require a multimodal approach to counter the complex cellular mechanisms that direct disease pathogenesis. In this study, we leveraged RNA-seq data to define genes that are suppressed in arthrofibrosis patients and identified adiponectin (*ADIPOQ*) as a potential candidate. We hypothesized that signaling pathways activated by ADIPOQ and the cognate receptors ADIPOR1 and ADIPOR2 may prevent fibrosis-related events that contribute to arthrofibrosis. (2) Methods: Therefore, ADIPOR1 and ADIPOR2 were analyzed in a TGFβ1 inducible cell model for human myofibroblastogenesis by both loss- and gain-of-function experiments. (3) Results: Treatment with AdipoRon, which is a small molecule agonist of ADIPOR1 and ADIPOR2, decreased expression of collagens (*COL1A1*, *COL3A1*, and *COL6A1*) and the myofibroblast marker smooth muscle α-actin (ACTA2) at both mRNA and protein levels in basal and TGFβ1-induced cells. (4) Conclusions: Thus, ADIPOR1 and ADIPOR2 represent potential drug targets that may attenuate the pathogenesis of arthrofibrosis by suppressing TGFβ-dependent induction of myofibroblasts. These findings also suggest that AdipoRon therapy may reduce the development of arthrofibrosis by mediating anti-fibrotic effects in joint capsular tissues.

## 1. Introduction

Arthrofibrosis is an incompletely characterized type of fibrosis that emerges after intra-articular trauma or surgical intervention. Arthrofibrosis may result after total knee arthroplasty (TKA) [1,2], and leads to debilitating limitations in joint range of motion with resultant pain, reduction in quality of life, and possible need for reoperation and/or revision TKA [3]. Aggregate data suggest that 4% of patients will develop arthrofibrosis after recovering from an otherwise well-fixed, well-aligned, and non-infected TKA [1,2,3,4].

Currently, non-surgical treatment options provide only modest improvements in clinical outcomes and are limited to physical therapy and manipulation under anesthesia (MUA). Moreover, only modest improvements are noted with revision TKAs. As such, it would be a major advancement in the practice of knee arthroplasty if it were possible to identify and prophylactically treat individuals at risk for arthrofibrosis [1,2,3,4].

The cellular and molecular mechanisms of arthrofibrosis share features with the pathogenesis of other pro-fibrotic diseases. Similarities include myofibroblasts as the primary effector cells, dysregulation of extracellular matrix formation with excessive collagen deposition, as well as alterations in inflammation-related tissue repair cascades [5,6,7]. Besides multiple molecular pathways involved in the development of arthrofibrosis, effective treatments for arthrofibrosis may require a multimodal approach to counter the complex cellular mechanisms that direct disease pathogenesis.

Therefore, we interrogated RNA-seq data to define novel genes that are differentially expressed in joint capsular tissues of arthrofibrosis patients. In this study, we show that adiponectin (*ADIPOQ*) and other adipogenic genes are selectively suppressed in arthrofibrotic tissues [8]. ADIPOQ is one of many adipokines that supports physiological homeostasis of adipose tissue, as well as other tissues and cell types, including macrophages, lymphocytes, endothelial, and epithelial cells. ADIPOQ contributes to regulation of systemic metabolism and insulin sensitivity, as well as anti-inflammatory processes [9,10]. Since ADIPOQ signals through its cognate receptors ADIPOR1 and ADIPOR2, we addressed the hypothesis that the ADIPOQ/ADIPOR1-2 signaling axis may control the phenotype of fibroblastic cells and the expression of genes involved in myofibroblastogenesis.

## 2. Materials and Methods

### 2.1. RNA-Sequencing Analysis

RNA-Seq analysis was recently performed on total RNA derived from posterior capsule knee tissues of arthrofibrotic and non-arthrofibrotic patients (GSE135854) [8]. These existing data were used to assess differentially expressed genes (i.e., either up- or downregulated) in arthrofibrotic and primary TKA tissues that were rendered visually as functional protein–protein interaction networks (STRING Database version 10.5) [11] and heat maps (Morpheus, Broad Institute) [12].

### 2.2. Cell Culture

We used a cell culture model for myofibroblastogenesis using mesenchymal stem/stromal cells (MSCs). These MSCs were derived from lipoaspirates obtained from consenting healthy donors using IRB approved protocols. We examined three representative donors (i.e., 211, 258, and 283), which have been previously characterized by RNA-sequencing and validated for standard stem cell markers, normal cell growth characteristics, and tri-lineage differentiation potential [13,14,15,16,17]. These MSCs are routinely cultured under zoonotic-free conditions using human platelet lysate (Mill Creek, Rochester, MN, USA) and are similar to MSCs used in clinical trials. MSCs in this study were used at passage 6. Prior to each experiment, MSCs at passage 5 were cultured in Advanced Minimal Essential Medium (Gibco/Thermo Fisher Scientific, Waltham, MA, USA) supplemented with 5% human platelet lysate (PLTMax, MillCreek Life Sciences, Rochester, MN, USA), 1% penicillin/streptomycin, 1% Glutamax (Gibco/Thermo Fisher Scientific, Waltham, MA, USA), and 0.2% heparin (Baxter, Deerfield, IL, USA). Cells were maintained in T175 flasks at 37 °C, 95% humidity, and 5% CO_2_ until they reached 80% confluency. Cells were then detached from T175 flasks by trypsinization using TrypLE Express (Gibco/Thermo Fisher Scientific, Waltham, MA, USA) and expanded into multi-well plates to obtain experimental replicates for different biological assays.

### 2.3. Immunofluorescence Microscopy

Cells grown on coverslips were fixed in 3.7% paraformaldehyde solution in phosphate-buffered saline (PBS) for 10 min at room temperature. Cells were permeabilized in 0.5% *v/v* Triton™ X-100 for 15 min, blocked for 1 h in 10% horse serum in PBS, and incubated with ACTA2 (α-SMA) antibody (Monoclonal Anti-Actin, α-Smooth Muscle—FITC antibody produced in mouse; Sigma Aldrich, St. Louis, MO, USA) at a 1:200 dilution overnight. The following day, cells were washed 3 times for 5 min in PBS with 0.5% *v/v* Triton™ X-100 before being incubated with a 1:1000 dilution of the secondary antibody (Goat anti-Mouse IgG2a Cross-Adsorbed Secondary Antibody, Alexa Fluor 488, Sigma Aldrich, St. Louis, MO, USA) for 1 h at room temperature in the dark. Cells were then washed again 3 times for 5 min in PBS and mounted in Texas Red™-X Phalloidin (Gibco/ThermoFisher Scientific, Waltham, MA, USA) and Fluoroshield DAPI (Sigma-Aldrich, St. Louis, MO, USA). Images were captured on a Zeiss Axio Vert.A1 microscope with a Zeiss AxioCam ICc 5 digital camera using a 10× objective.

### 2.4. RNA Interference

*ADIPOR1* and *ADIPOR2* siRNA smart pools, as well as non-silencing control siRNAs were purchased from Dharmacon (GE Healthcare, Lafayette, CO, USA). Transfections were carried out as recommended by the manufacturer. Cells were transfected with siRNA (10 nM) in serum-free Opti-MEM (Gibco/ThermoFisher Scientific, Waltham, MA, USA) for 6 h and then replaced with normal culture medium (see above). Cells were harvested and processed for MTS activity and RNA extraction 48 and 72 h after transfection, respectively.

### 2.5. MTS Assay

Colorimetric assays measuring conversion of (3-(4,5-dimethylthiazol-2-yl)-5-(3- carboxymethoxyphenyl)-2-(4-sulfophenyl)-2H-tetrazolium inner salt (MTS-assays) were used to monitor cellular metabolic activity as an indicator of cell viability, proliferation and cytotoxicity according to the manufacturer’s protocol (Promega, Madison, WI, USA). Absorbance was measured at 490 nm using a SpectraMAX Plus spectrophotometer (Molecular Devices, San Jose, CA, USA).

### 2.6. AdipoRon Treatment During TGFβ1 Induction of Myofibroblast Formation

AdipoRon treatments were performed with MSCs that were either maintained at a basal state or differentiated by TGFβ1 administration. AdipoRon (Tocris, Minneapolis, MN, USA) was prepared as a 25 mM stock solution in dimethylsulfoxide (DMSO). The dosing of AdipoRon was analyzed using MTS assays to ensure that the selected dose (50 nM) is not cytotoxic for MSCs. Lyophilized, human TGFβ1 was purchased from R&D Systems (Minneapolis, MN, USA) and reconstituted at 10 µg/mL with sterile 4 mM HCl containing at least 0.1% bovine serum albumin according to the manufacturer’s instructions. Cells were treated in the presence or absence of TGFβ1 (10 ng/mL) with either vehicle (DMSO) or AdipoRon (25 µM). We generated four treatment groups in which cells were analyzed in the absence of TGFβ1 with or without AdipoRon (groups 1 and 2) or treated in the presence of TGFβ1 with or without AdipoRon (groups 3 and 4). Cells were treated on both Day 0 (80% confluency) and Day 4. Cells were harvested for RNA and protein extraction at Day 7.

### 2.7. Analysis of Gene Expression

RNA was isolated from cultured MSCs in which ADIPOR1 and ADIPOR2 functions were modulated to monitor the expression of relevant biomarkers. RNA was extracted using the miRNeasy mini kit (Qiagen, Germantown, MD, USA) according to the manufacturer’s protocol. All RNA samples were immediately lysed using TRIzol, which inactivates RNAases, prior to column purification. RNA was quantified using a Nanodrop 2000 spectrophotometer (Gibco/Thermo Fisher Scientific, Waltham, MA, USA). A260/280 ratios of >2 confirmed the absence of DNA or protein contamination in our samples. RNA was also routinely analyzed for RNA integrity number (RIN) scores and values are typically between 9 and 10.

Reverse transcription reactions were performed using 1000 ng of total RNA. Each RNA sample was diluted with RNAse free H_2_O to a total of 9.5 µL in a 96 well PCR plate. In a 1.5 mL micro-centrifuge tube, the following reagents were mixed to generate a master mix sufficient for all reactions (‘reaction 1 mixture’; volumes indicated are for a single PCR reaction): 1 µL Random DNA Primers (50 ng/µL) (Promega), 2 µL dNTPs (2.5 mM) (Sigma, St. Louis, MO, USA) and 0.5 µL RNasin (Promega). Reaction 1 mixture (3.5 µL) was added to the RNA samples present in each 96 PCR well plate and incubated at 65 °C for 3 min to support annealing. In another 1.5 mL micro-centrifuge tube, the following reagents were mixed to generate a second master mix sufficient for all reactions (‘reaction 2 mixture’; volumes indicated are for a single PCR reaction): 2 µL DTT, 4 µL 5X buffer and 1 µL MLV-RT (Promega). Reaction 2 mixture (7 µL) was added to each annealed DNA/RNA sample on the 96 well PCR plate. The plate was run in a thermal cycler (Biorad, Hercules, CA, USA) at the following cycling temperatures: 95 °C for 10 min, followed by 37 °C for 90 min. The resulting cDNA was diluted (8 ng/µL) in RNase/DNase free water and stored at −20 °C.

Real time reverse transcriptase quantitative PCR (RT-qPCR) reactions were performed using unmodified DNA oligonucleotides and SYBR Green (Qiagen) detection and amplification of (18 to 21 bp to match melting temperatures). Oligonucleotide primer pairs were either previously published (e.g., *GAPDH*, *COL1A1*) or designed using the NCBI BLAST program (e.g., *ADIPOR1*, *ADIPOR2*). RT-qPCR reactions were performed using 2.5 µL gene-specific primers (1 pmol) (Invitrogen, Carlsbad, CA) (Supplementary Materials Table S1), 2.5 µL cDNA (20 ng) and 5 µL SYBR Green combined for each reaction in a 384 well PCR plate (CFX384 Real-Time System (BioRad, Hercules, CA, USA)). PCR was performed using the following cycle conditions: initial denaturation at 95 °C for 10 min, followed by 40 cycles at 95 °C for 2.15 min, 60 °C for 0.45 min, and 65 °C for 0.05 min.

Transcript levels were quantified using the ΔΔCt method and normalized to the housekeeping gene *GAPDH* (set at 100). Saturation of the melting curves is typically observed between 17 and 30 cycles for all mRNAs we tested. While we would disregard any samples with highly aberrant *GAPDH* cycle numbers above an arbitrary threshold (>20), we note that all samples we tested in our studies have *GAPDH* cycle numbers between 17 and 19. Three donors were analyzed as three biological replicates and tested as technical duplicates by RT-qPCR. Gene expression values were determined in batches where all genes were simultaneously tested for each donor.

### 2.8. Protein Extraction and Immunoblotting

Protein lysates were collected by scraping cells with 70 µL of radio-immunoprecipitation assay (RIPA) buffer supplemented with 5 mM ethylene diamine tetraacetic acid (EDTA) and 1X protease inhibitor cocktail (Gibco/ThermoFisher Scientific, Waltham, MA, USA). Samples were subjected to 30 min of intermittent vortexing (30 s) and then centrifuged at 12,000× *g* for 15 min at 4 °C to remove cellular debris. Protein concentrations were determined utilizing the DC Protein Assay Kit II (Bio-Rad, Hercules, CA, USA). A total of 15 μg of each sample was resolved via SDS-PAGE and successively transferred to a nitrocellulose membrane. All membranes were then blocked for 1 h at room temperature in StartingBlock™ T20 (TBST 0.1%, Gibco/ThermoFisher Scientific, Waltham, MA, USA), placed into primary antibody overnight at 4 °C, and subsequently placed into secondary antibody for 1 h at room temperature. All proteins were visualized using the SuperSignal™ West Pico PLUS Chemiluminescent Substrate kit, and final images were captured using a Bio-Rad ChemiDocTM Touch Imaging System. Primary antibodies used were: ACTA2 (1:1000; ab21027; Abcam, Cambridge, MA, USA) and GAPDH (1:30,000; ab181602; Abcam, Cambridge, MA, USA).

### 2.9. Statistical Analysis

GraphPad Prism version 7.03 for Windows was used to create graphs and perform statistical analysis. Continuous variables were analyzed with t-test and one-way ANOVA. When applicable, significance is noted in the figures with a standard asterisk convention: *p* ≤ 0.0001 = ****; *p* ≤ 0.001 = ***; *p* ≤ 0.01 = **; *p* ≤ 0.05 = *; *p* > 0.05 = not significant (ns).

## 3. Results

**Figure 1:**
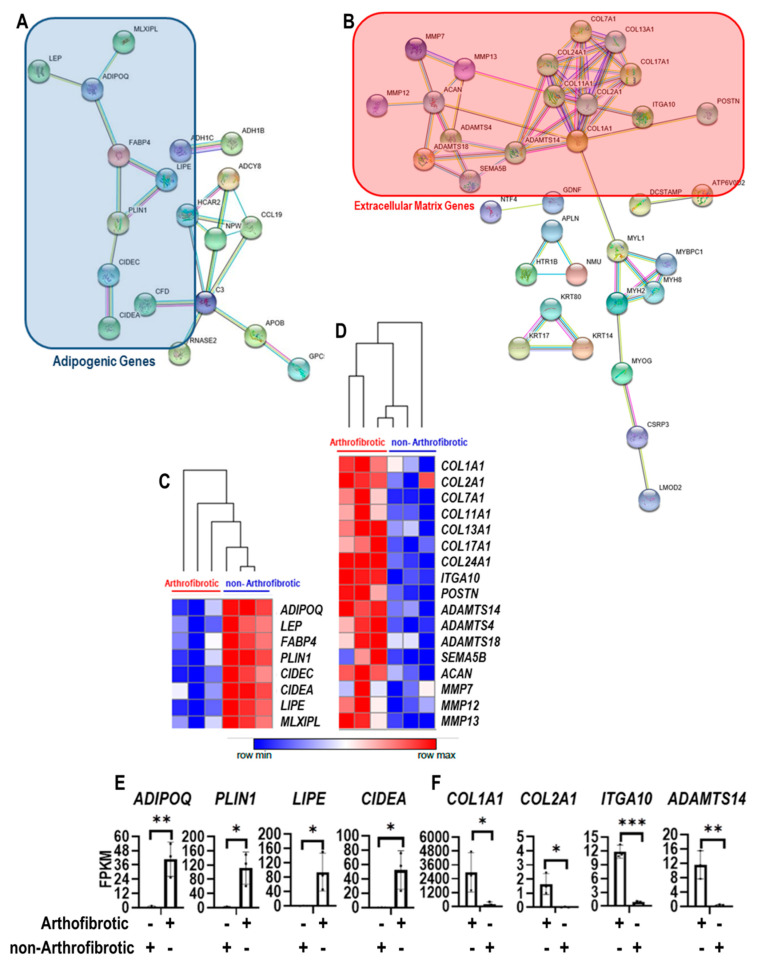
Protein–protein interaction networks, heat maps and expression levels of adipogenic and extracellular matrix genes identified by RNA-seq analysis arthrofibrotic and non-arthrofibrotic tissues. String analysis of differentially expressed genes in arthrofibrotic and non-arthrofibrotic tissues of RNA-seq data illustrates genes with high confidence interactions were grouped as downregulated adipogenic genes (in blue box) (**A**) and upregulated extracellular matrix genes (in red box) (**B**). The entire set of genes exhibiting minimally a 2-fold change in gene expression included 1795 downregulated and 2059 upregulated genes. To permit proper visualization, we arbitrarily selected only the Top 100 genes that exhibited the largest modulation in expression. String networks were rendered with settings at high confidence (0.700) and unconnected proteins were not visualized. Heatmaps of expression values in arthrofibrotic and non-arthrofibrotic tissues were also analyzed by Morpheus and clustered separately for adipogenic (**C**) and extracellular matrix (**D**) genes. Expression levels by RNA-seq are represented as fragments per kilobase of transcript per million (FPKM) of adipogenic (**E**) and extracellular matrix (**F**) genes in posterior capsule knee tissues derived from arthrofibrotic and non-arthrofibrotic patients. Statistical differences in expression levels between non-arthrofibrotic and arthrofibrotic samples are shown with brackets. Asterisks reflect the following probability values: *p* ≤ 0.001 = ***; *p* ≤ 0.01 = **; *p* ≤ 0.05 = *. Error bars represent the mean ± standard deviation (*n* = 3) for each arthrofibrotic and non-arthrofibrotic tissue. The three actual data points are shown as dots superimposed on the graphs.

Previous studies have revealed changes in gene expression between arthrofibrotic versus primary TKA tissues [8]. As an introductory illustration of these findings, which provide the experimental foundation of the present study, representative sets of differentially expressed genes in these patient groups were visualized as networks using STRING analysis. Differences in gene expression programs in tissues from arthrofibrotic versus primary TKA patients reflect a decreased expression of adipogenic genes (Figure 1A) and increased expression of fibroblast-related extracellular matrix proteins (Figure 1B). Select markers were also rendered as heat maps using Morpheus matrix visualization to highlight differential expression of adipogenic (Figure 1C) versus extracellular matrix genes (Figure 1D) in arthrofibrotic and non-arthrofibrotic tissues. Differential mRNA expression of well-established adipogenic (Figure 1E) and extracellular matrix (Figure 1F) genes is also illustrated by bar graphs, including data for the secreted adipogenic ligand ADIPOQ that provided the basis for a focus on the adiponectin receptors ADIPOR1 and ADIPOR2 in the present study. The error bars reflect both biological and technical variation in transcript levels among distinct surgical specimens due to differences in surgical isolation and sample processing, precise anatomical location, the distribution of cells and tissues within the specimens, and patient co-morbidities.

**Figure 2:**
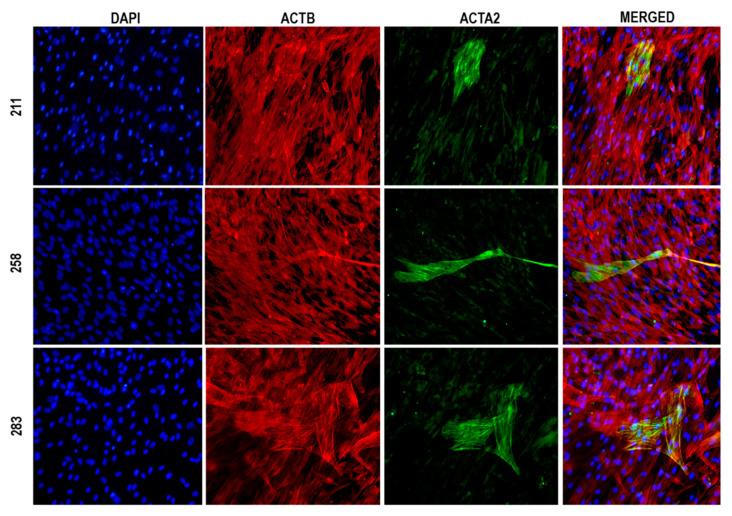
Pre-myofibroblastic nature of mesenchymal stem/stromal cells (MSCs). Immunofluorescence microscopy showing cellular morphology of MCSs from three donors (211, 258 and 283). The panels show DNA staining in the nucleus (DAPI, blue), as well as cytoskeleton staining of smooth muscle β-actin (ACTB; red) and smooth muscle α-actin (ACTA2; green). Modified ACTA2 stress fibers are apparent in a subset of cells that resemble the myofibroblast phenotype. Images were captured with a 10× objective.

*ADIPOQ* is among the most prominent genes with diminished gene expression in arthrofibrotic tissues based on RNA-seq data (Figure 1), and it has been functionally implicated in the molecular pathology of fibrosis [18,19,20,21]. We postulated that reduced expression of *ADIPOQ* in posterior capsule tissue may diminish paracrine signaling. Loss of *ADIPOQ* is predicted to impair the adiponectin/adiponectin receptor signaling pathway in arthrofibrotic tissues and could contribute to disease progression. Since ADIPOQ signals through its cognate receptors ADIPOR1 and ADIPOR2, we designed a series of loss and gain of function studies to elucidate the role of the ADIPOQ/ADIPOR1-2 signaling axis on metabolic activity and differentiation of fibroblastic progenitors into myofibroblasts. The morphological differentiation of immature fibroblasts into myofibroblast in fibrotic tissues occurs concomitantly with induction of smooth muscle α-actin (ACTA2), which is the main cytoskeletal marker of modified stress fibers in myofibroblasts. While many different cell types can be differentiated into ACTA2 positive myofibroblasts (e.g., fibroblasts, epithelial cells, smooth muscle cell, endothelial cells, pericytes) [22], our functional studies were performed using MSCs derived from the perivascular stroma of blood vessels in adipose tissue from otherwise normal donors as a human cell culture model of myofibroblastogenesis. In vivo, these MSCs are activated and recruited by platelets to form transient connective tissue for stress shielding of injured tissues. Therefore, MSCs are propagated in our cell culture model using human platelet lysate (rather than bovine serum as is customary) to approach the physiological environment of healing tissue. The pre-myofibroblastic nature of these perivascular MSCs was assessed by immunofluorescence microscopy. Indeed, immunofluorescence images show that MSCs spontaneously differentiate under basal conditions into myofibroblasts as measured by ACTA2 staining (Figure 2). We note that ACTA2 myofibroblasts assume an enlarged flattened morphology that is rather distinct from the narrow spindle-shaped morphology of ACTB positive fibroblasts that are imaged at the same level of magnification. Experiments were replicated in MSCs derived from three distinct donors (i.e., 211, 258 and 283).

**Figure 3:**
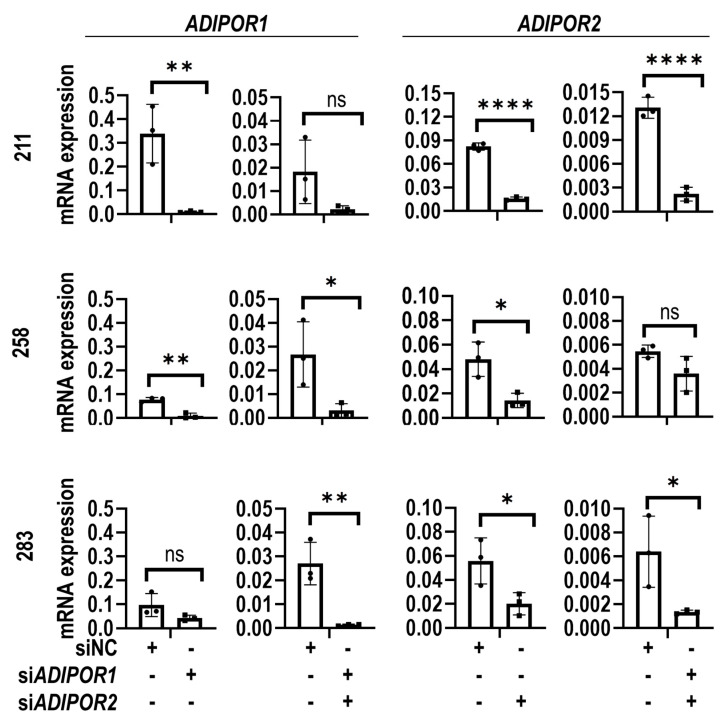
Depletion of adiponectin receptor 1 (*ADIPOR1*) and *ADIPOR2* in MSCs. RT-qPCR data show mRNA expression levels of *ADIPOR1* and *ADIPOR2* upon transfection with non-silencing control (siNC), or siRNAs for *ADIPOR1* (si*ADIPOR1*), *ADIPOR2* (si*ADIPOR2*) or the combination of *ADIPOR1* and *ADIPOR2* (si*ADIPOR1*+si*ADIPOR2*) in MSCs derived from three distinct donors (211, 258 and 283). Statistical difference in gene expression between groups is shown with brackets. Asterisks indicate differences in probability values: *p* ≤ 0.0001 = ****; *p* ≤ 0.01 = **; *p* ≤ 0.05 = *; *p* > 0.05 = not significant (ns). Statistical significance and error bars (mean ± standard deviation) are based on *n* = 3 biological replicates for each of the three MSC donors (data points superimposed on the graphs).

Depletion of *ADIPOR1* and *ADIPOR2* mRNAs using gene-specific siRNAs was validated by RT-qPCR (Figure 3). A significant decrease in *ADIPOR1* and *ADIPOR2* gene expression was observed in cells from all three donors (i.e., 211, 258, 283), but these values only reached statistical significance in a subset of the samples. This observation is likely due to variation in biological replicates.

**Figure 4:**
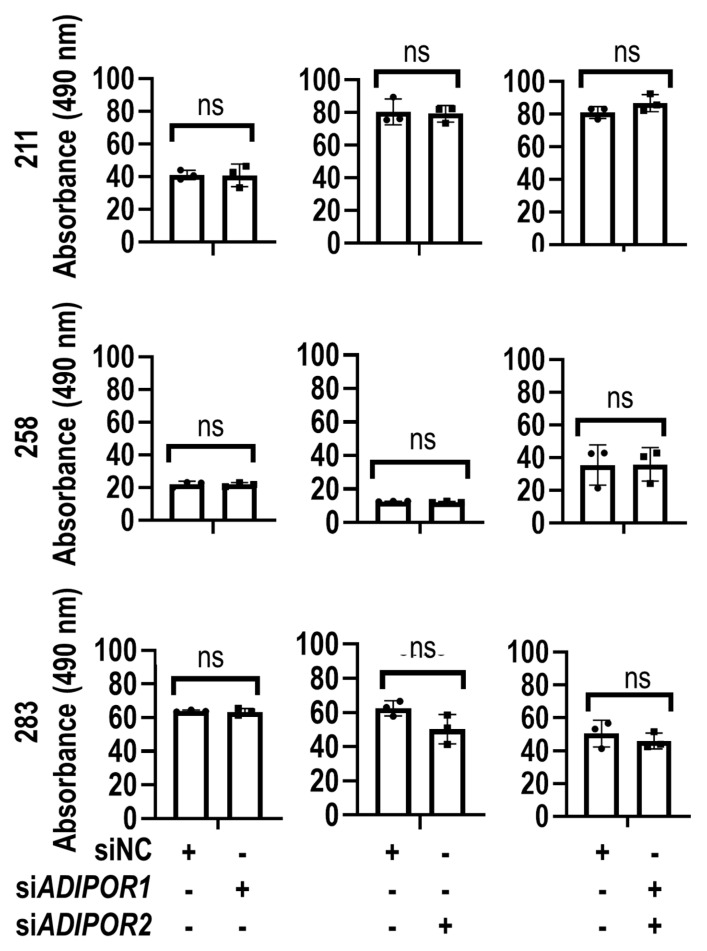
Metabolic activity is preserved in MSCs lacking *ADIPOR1* and *ADIPOR2* expression. Cellular metabolic activity was assessed by MTS assay in MSCs derived from three donors (211, 258 and 283), transfected with (siNC), or siRNAs for *ADIPOR1* (si*ADIPOR1*), *ADIPOR2* (si*ADIPOR2*) or the combination of *ADIPOR1* and *ADIPOR2* (si*ADIPOR1*+si*ADIPOR2*). Statistical difference in gene expression between groups is shown with brackets. *p* > 0.05 = not significant (ns). Statistical significance and error bars (mean ± standard deviation) are based on *n* = 3 biological replicates for each of the three MSC donors (data points superimposed on the graphs).

The biological effects of si*ADIPOR1* and si*ADIPOR2* treatment on the metabolic activity of MSCs were examined by MTS assays. The results show that transfection of *ADIPOR1* and *ADIPOR2* siRNAs did not have appreciable effects on cellular metabolic activity (Figure 4).

**Figure 5:**
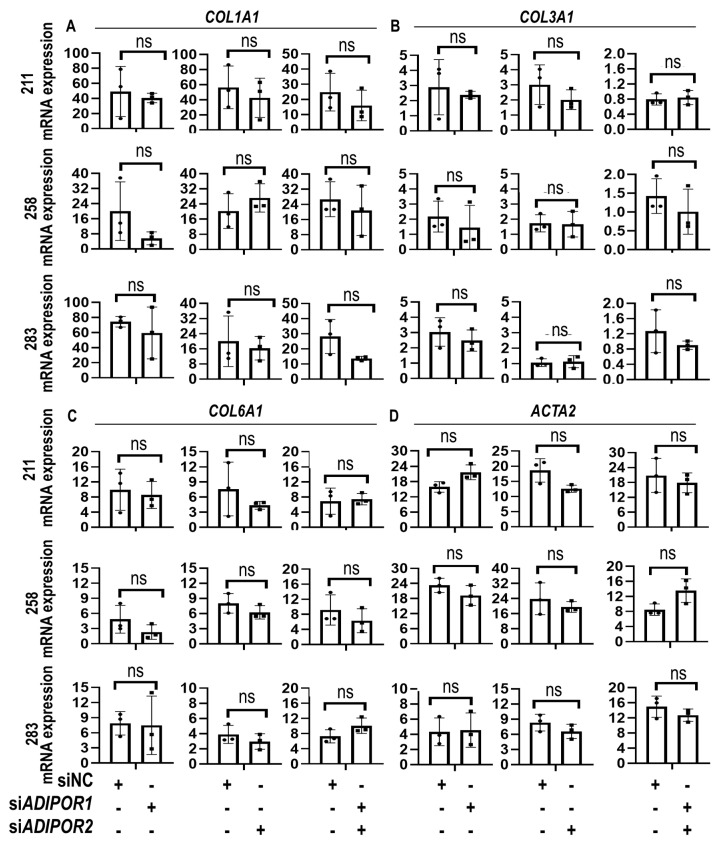
Depletion of ADIPOQ receptors preserves expression of fibrosis-related markers. Gene expression data obtained by RT-qPCR for fibrosis-related genes *COL1A1* (**A**), *COL3A1* (**B**), *COL6A1* (**C**), and *ACTA2* (**D**) after transfection with non-silencing control (siNC), or siRNAs for *ADIPOR1* (si*ADIPOR1*), *ADIPOR2* (si*ADIPOR2*) or the combination of *ADIPOR1* and *ADIPOR2* (si*ADIPOR1* + si*ADIPOR2*) in MSCs derived from three distinct donors (211, 258 and 283). Statistical difference in gene expression between groups is shown with brackets. Asterisks indicate differences in probability values: *p* > 0.05 = not significant (ns). Statistical significance and error bars (mean ± standard deviation) are based on *n* = 3 biological replicates for each of the three MSC donors (data points superimposed on the graphs).

Subsequent RT-qPCR analysis of representative collagens (*COL1A1*, *COL3A1*, and *COL6A1*) and the myofibroblast marker *ACTA2* indicated that siRNA mediated depletion of *ADIPOR1* and *ADIPOR2* did not modulate expression of these fibrosis-related mRNAs (Figure 5A–D). Together, these results indicated that the functions of ADIPOR1 and ADIPOR2 under normal conditions are dispensable for maintaining a basal level of metabolic activity or expression of fibrotic genes.

**Figure 6:**
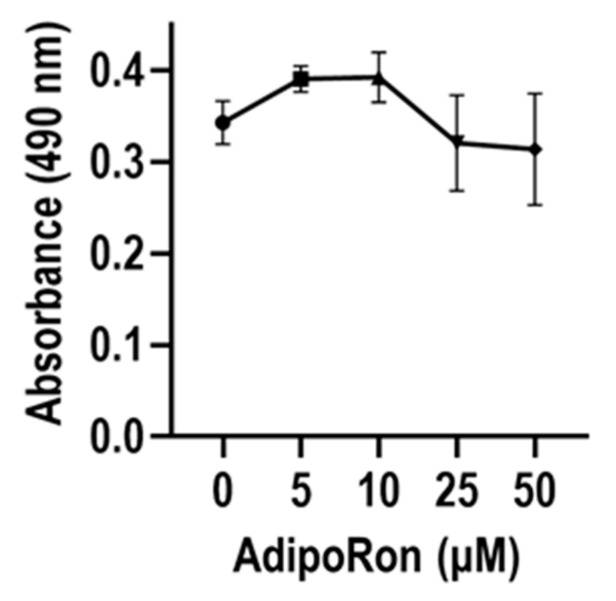
Dose optimization of the adiponectin receptor agonist AdipoRon by MTS assay. The results show that concentrations of up to 50 µM of AdipoRon do not statistically affect cell metabolism in MSCs derived from three distinct donors (211, 258 and 283). Line graphs show combined results for three biological replicates for each of the three donors (mean ± standard deviation).

To test whether activation of ADIPOR1 and/or ADIPOR2 mediated signaling pathways contributes to myofibroblastogenesis, we examined the cellular effects of the ADIPOQ receptor agonist—AdipoRon—on gene expression. Titration experiments revealed that AdipoRon does not affect cell metabolic activity at doses of 50 µM or less in MSCs as measured by MTS assays (Figure 6).

**Figure 7:**
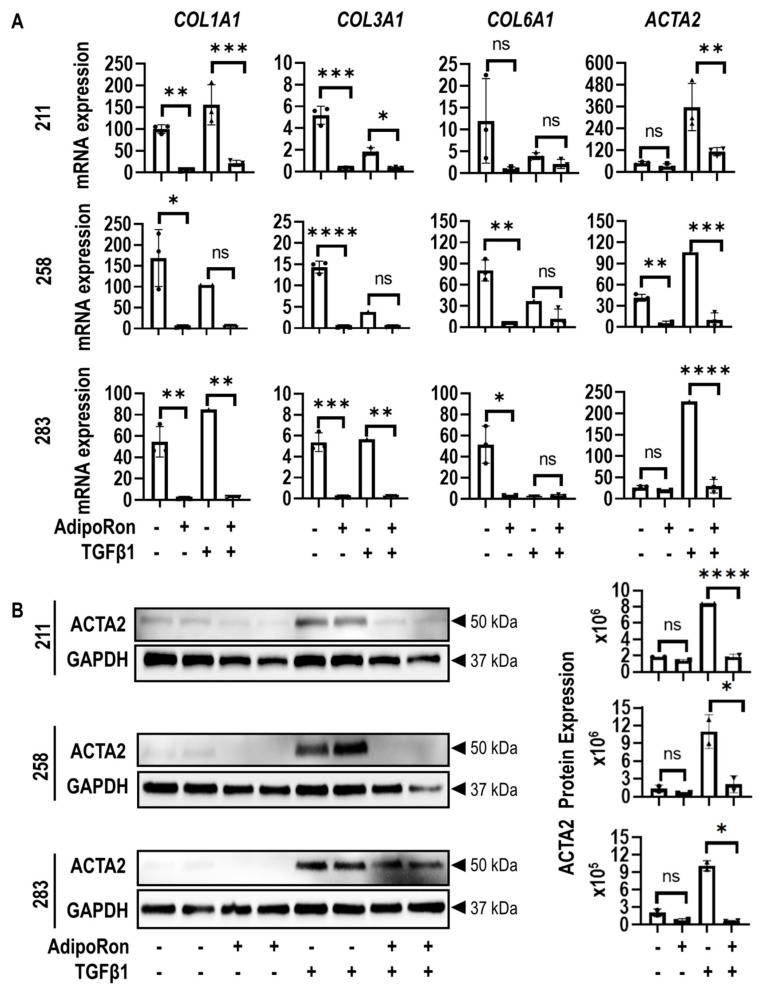
AdipoRon suppresses expression of fibrotic-related markers in MSCs. (**A**) Gene expression data obtained by RT-qPCR for fibrosis-related collagen genes *COL1A1*, *COL3A1*, *COL6A1*, and *ACTA2*) in MSCs from three different MSC donors (211, 258, and 283) non-treated and treated with AdipoRon in the presence or absence of TGFβ1. (**B**) Western blotting of three MSCs donors non-treated and treated with AdipoRon in the presence or absence of TGFβ1. The data show that TGFβ1 induces ACTA2 levels and that AdipoRon inhibits this TGFβ1-dependent induction. Statistical difference in gene expression between groups is shown with brackets. Asterisks indicate differences in probability values: *p* ≤ 0.0001 = ****; *p* ≤ 0.001 = ***; *p* ≤ 0.01 = **; *p* ≤ 0.05 = *; *p* > 0.05 = not significant (ns). Statistical significance and error bars (mean ± standard deviation) are based on *n* = 3 biological replicates for each of the three MSC donors (data points superimposed on the graphs). Western blot quantitation shows results for *n* = 2 biological replicates for each of the three MSC donors. Error bars present the mean ± range of the two biological replicates (represented as 2 dots superimposed on the graph).

MSCs were then treated for two cycles with AdipoRon (25 µM) to assess effects on ADIPOQ receptor activation under basal and TGFβ1 conditions. Samples were analyzed by RT-qPCR assays to assess mRNA levels and by western blot analyses to define protein levels. MSCs treated with AdipoRon showed a significant decrease in the myofibroblast marker ACTA2 in TGFβ1 treated cells at both mRNA (Figure 7A) and protein levels (Figure 7B). AdipoRon also reduced the expression of *COL1A1* and *COL3A1* mRNAs (Figure 7A), but these changes on gene expression appear to be donor-dependent.

**Figure 8:**
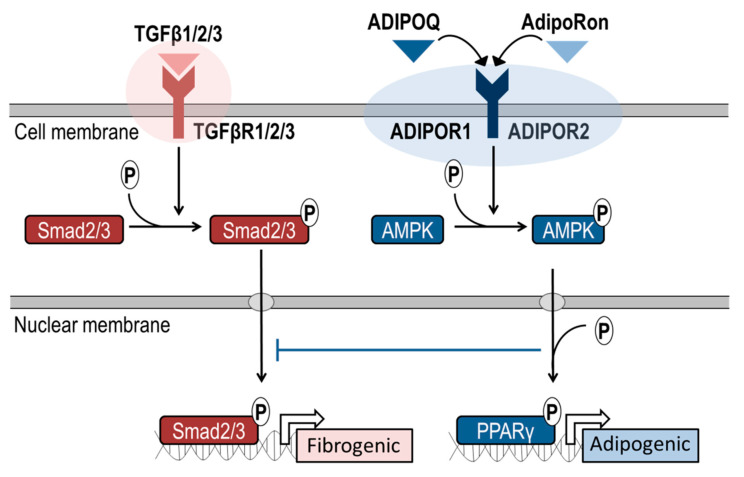
Model depicting pharmacologic modulation of adipogenic pathways for treatment of arthrofibrosis. Schematic showing intra-cellular signaling cascades of TGFβ mediated pro-fibrotic pathways and proposed ADIPOR1- and ADIPOR2-related pathways that inhibit TGFβ dependent molecular pathways. Canonical TGFβ signaling is characterized by a SMAD2/3 initiated cascade leading to inflammation and extracellular matrix production. Parallel activation of the ADIPOR1 and/or ADIPOR2 pathways (blue box) by AdipoRon or ADIPOQ results in downregulation of inflammatory transcription factors and upregulation of PPARγ—a primary transcription factor regulating adipogenic differentiation.

The reduced expression of ACTA2 by AdipoRon is consistent with a model in which ADIPOQ receptor signaling suppresses human myofibroblastogenesis by interfering with the development of a pro-fibrotic state that is promoted by canonical TGFβ signaling and concomitant activation of SMAD transcription factors [23]. This model clarifies how AdipoRon treatment can decrease both collagen levels and the myofibroblast marker ACTA2, as well as provides a mechanism-based strategy by which AdipoRon may mitigate progression of arthrofibrosis (Figure 8).

## 4. Discussion

The present study addressed whether the adipogenesis-related proteins ADIPOR1 and ADIPOR2 contribute to fibrotic disease progression, which would identify these receptors as potential drug targets for arthrofibrosis. Previous studies suggest a modulatory role for adipokines in the development of fibrosis in various organs [18,19,20,21]. ADIPOQ is a known adipokine with anti-fibrotic effects and its activity is mediated through the transmembrane receptors ADIPOR1 and ADIPOR2 [24]. The findings in our study suggests that targeting of ADIPOQ receptors represents a viability strategy for attenuating myofibroblastogenesis—a biological process that supports the arthrofibrosis cascade.

Myofibroblasts have long been recognized as the primary mediators of fibrogenesis in both early and late fibrotic disease pathways [23,25,26]. Recent studies have highlighted the multiplicity of origins and roles of myofibroblasts, as well as the unique responses they exert based upon their local environmental conditions and location in the body [6,27,28,29]. The complexity of fibrogenesis has been further elucidated by results describing the intricate cross-talk amongst fibrogenic, adipogenic, and myogenic pathways [30]. All of these different molecular pathways involved in arthrofibrosis disease progression propose that effective treatments for arthrofibrosis may require a multimodal approach to counter the complex cellular mechanisms that direct disease pathogenesis. The identification of the adipogenesis-related receptors, ADIPOR1 and ADIPOR2, as arthrofibrosis-related molecules provides further insight into the importance of the adipogenesis pathways in fibrotic disease progression. Additionally, the ability of AdipoRon to decrease myofibroblastic markers by activating ADIPOQ receptors (i.e., ADIPOR1 or ADIPOR2) in cell-based experiments reflects the potential utility of ADIPOR1 and ADIPOR2 as therapeutic targets.

The primary endogenous agonist of ADIPOR1 and ADIPOR2 is ADIPOQ—the most abundant adipokine produced by adipocytes. AdipoRon is a novel orally active small molecule that binds to the adiponectin receptors ADIPOR1 and ADIPOR2 to activate AMPK kinase activity and transcription factor PPARγ, respectively. This compound induces several favorable metabolic effects, including improved insulin sensitivity, weight neutrality, and expanded life span. Recent studies also revealed that AdipoRon exerts protective effects in preclinical models of pulmonary, cardiac, and hepatic fibrosis [21,31,32,33]. PPARγ has complex, pleiotropic cellular effects. In the context of fibrosis, PPARγ sets the pre-adipocytes on a path of adipogenic differentiation with upregulation of ADIPOQ in a feed-forward cycle. For macrophage lineage cells, PPARγ activation results in biasing the cells to differentiate to an M2 phenotype—their anti-inflammatory state. M2 macrophages promote decreased tissue infiltration and increase tissue repair [34]. Although PPARγ is a major regulator of adipogenesis, it does not act independently, and its adipogenic effects are intimately associated with and inversely proportional to ADIPOQ levels [35,36]. In view of the possible role of ADIPOR1 and ADIPOR2 in arthrofibrosis, which is suggested by findings in this study, the relationship between PPARγ and ADIPOQ provides potential options for pharmacologic modulation of adipogenic pathways in arthrofibrosis.

AdipoRon is not the first pharmacologic mediator of adipogenesis leveraged for anti-fibrotic effects. Thiazolidinediones (TZDs), a class of drugs traditionally used for diabetic treatment, have reduced fibrotic disease burden in multiple pathogenic states. TZDs (e.g., rosiglitazone) act as direct agonists of PPARγ and have shown reduction of fibrotic disease when administered in vivo in animal models of pulmonary and joint fibrosis [37,38,39,40].

The putative anti-fibrotic effects of adipogenic agents have not yet been observed or investigated to the same degree in clinical practice, preventing translation of this viable concept into therapy. Alteration of adipogenesis homeostasis may be responsible for this observation as ADIPOQ levels are reported to be significantly decreased in patients affected by fibrotic processes in cardiopulmonary, renal, and hepatic systems [41,42,43]. In the absence of ADIPOQ-activated cellular signaling, PPARγ expression is decreased, thereby modulating the therapeutic effects of TZDs [36,41]. Loss of ADIPOQ is predicted to reduce both adipogenic cell signaling and the levels of PPARγ. Since TZDs are ligands for PPARγ, loss of PPARγ could potentially desensitize cells to TZDs. Conversely, increased adipogenic signaling by AdipoRon, which acts on ADIPOR1 and ADIPOR2, is expected to elevate PPARγ and render cells more sensitive to TZDs.

AdipoRon offers an alternative mechanism of achieving PPARγ activation through direct agonistic activity of ADIPOQ receptors (i.e., ADIPOR1 or ADIPOR2). Therefore, AdipoRon therapy is hypothesized to be less affected by alterations in adipogenic homeostasis (i.e., reductions in ADIPOQ levels). This notion is supported by the efficacy of AdipoRon when implemented in other fibrotic diseases [44,45,46]. Interestingly, in a liver model of fibrosis, ADIPOR2 was established as a primary receptor that is responsible for mediating the anti-fibrotic effects of ADIPOQ in vivo [45].

The present work has identified ADIPOR1 and ADIPOR2, which are two adipogenesis-related receptors, as potential drug targets of interest in arthrofibrosis and further demonstrated the anti-fibrotic effects of AdipoRon in vitro. Importantly, this study and others have emphasized the diverse nature of myofibroblastogenesis and the potential utility of pharmacologic targets along the adipogenesis pathway. Since the fibrotic process shares similar cellular and molecular pathologies in different tissues, findings and translational implications from the present study may also apply to fibrosis in other musculoskeletal tissues and joints (e.g., shoulder, hip). Future in vivo studies exploring specific drug delivery mechanisms, dosing, and tissue-specific effects are required to evaluate the role of AdipoRon as a viable therapy for arthrofibrosis.

## Supplementary Materials

The following are available online at https://www.mdpi.com/2077-0383/9/11/3690/s1, Table S1: Gene-specific primers used for analysis of gene expression.
