# Supplementary material for: A Potential Theragnostic Regulatory Axis for Arthrofibrosis Involving Adiponectin (ADIPOQ) Receptor 1 and 2 (ADIPOR1 and ADIPOR2), TGFβ1, and Smooth Muscle α-Actin (ACTA2)
Source: J Clin Med. 2020 Nov 17;9(11):3690. doi: 10.3390/jcm9113690 (PMC7698546; doi:10.3390/jcm9113690)
Supplement: Supplementary file 1 [file jcm-09-03690-s001.pdf]

Supplementary Materials Table S1. Gene specific primers used for analysis of gene expression.

Gene Symbol	Primer Orientation	Nucleotide Sequence (5′-3′)	Product Size (bp)	Cycles (range)
ADIPOR1	Forward	CCTGCCCGGCTTGTCTAC	208	27–32
	Reverse	CCCGTTTGCCCTTCTCTTCT		
ADIPOR2	Forward	GACACGCGGATCAACTCACT	153	30–35
	Reverse	GTTGGTGCCCTTTTCTGAGC		
COL1A1	Forward	GTAACAGCGGTGAACCTGG	98	18–28
	Reverse	CCTCGCTTTCCTTCCTCTCC		
COL3A1	Forward	TTGAAGGAGGATGTTCCCATCT	60	21–29
	Reverse	ACAGACACATATTTGGCATGGTT		
COL6A1	Forward	ATTGCCAAGGACTTCGTCGT	173	19–25
	Reverse	TCCACTGCAGGCTCTTGATG		
ACTA2	Forward	AAAAGACAGCTACGTGGGTGA	54	20–22
	Reverse	GCCATGTTCTATCGGGTACTTC		
GAPDH	Forward	ATGTTCGTCATGGGTGTGAA	124	17–25
	Reverse	TGTGGTCATGAGTCCTTCCA		
